# Disinfection of Human and Porcine Corneal Endothelial Cells by Far-UVC Irradiation

**DOI:** 10.3390/medicina61030416

**Published:** 2025-02-27

**Authors:** Ben Sicks, Martin Hessling, Kathrin Stucke-Straub, Sebastian Kupferschmid, Ramin Lotfi

**Affiliations:** 1 Institute of Medical Engineering and Mechatronics, Ulm University of Applied Sciences, Albert-Einstein-Allee 55, 89081 Ulm, Germany; martin.hessling@thu.de (M.H.); kathrin.stucke-straub@thu.de (K.S.-S.); 2 Clinic of Ophthalmology, Bundeswehrkrankenhaus Ulm, Oberer Eselsberg 40, 89081 Ulm, Germany; sebastiankupferschmid@bundeswehr.org; 3 Institute for Clinical Transfusion Medicine and Immunogenetics Ulm, German Red Cross Blood Donation Service Baden-Württemberg-Hessen, Institute for Transfusion Medicine, University Hospital Ulm, Helmholtzstraße 10, 89081 Ulm, Germany; r.lotfi@blutspende.de

**Keywords:** cornea irradiation, donor, transplant, human eye, 222 nm Far-UVC

## Abstract

*Background and Objectives*: The cornea protects the eye from external influences and contributes to its refractive power. Corneas belong to the most frequently transplanted tissues, providing a last resort for preserving the patient’s vision. There is a high demand for donor corneas worldwide, but almost 4% of these transplants are not eligible due to microbial contamination. The objective of this study is to ascertain the suitability of 222 nm Far-UVC irradiation for the decontamination of corneas without damaging corneal endothelial cells. *Materials and Methods*: To assess the destructive effect of irradiation and, thus, identify the applicable dose needed to decontaminate the cornea without interfering with its integrity, 141 porcine corneas were irradiated with 0, 60 or 150 mJ/cm^2^ at 222 nm. In the second step, a series of 13 human corneas were subjected to half-sided irradiation using 15 or 60 mJ/cm^2^ at 222 nm. After five days of in vitro culturing, the endothelial cell density of the non-irradiated area of each human cornea was compared to the irradiated area. *Results*: Irradiation with up to 60 mJ/cm^2^ had no detectably significant effect on the cell integrity of human corneas (*p* = 0.764), with only a minimal reduction in cell density of 3.7% observed. These findings were partially corroborated by tests on porcine corneas, wherein the variability between test groups was consistent, even at increased irradiation doses of up to 150 mJ/cm^2^, and no notable effects on the irradiated porcine endothelium were monitored. The efficacy of the antimicrobial treatment was evident in the disinfection tests conducted on corneas. *Conclusions*: These initial irradiation experiments demonstrated that 222 nm Far-UVC radiation has the potential to decontaminate the cornea without compromising sensitive endothelial cell viability.

## 1. Introduction

Corneas are commonly known as the visual gateway to the world due to their optical properties [1,2]. Various serious diseases or injuries, which impact corneal integrity, can lead to vision loss [3,4,5,6,7]. In such cases, keratoplasty is often considered as the last option to save eyesight. Annually, over 200,000 corneas are transplanted worldwide [8]. For a keratoplasty, donor corneas are needed, which must fulfil various quality criteria [9]. Corneal transplants need to be free of pathogens and to have a sufficient density of endothelial cells [10]. Corneal endothelial cells (CECs) are in charge of maintaining corneal clarity by preventing the cornea from becoming edematous [11,12]. Unfortunately, CECs are very sensitive to chemical and physical stress and do not have regenerative capacities, which leads to the continuous loss of their number during life span [1,11,13,14]. This results in the exclusion of a considerable proportion of tissue donations not fulfilling minimal criteria for endothelial cell density. In addition, bacterial contamination occurs in some donations, which, in turn, leads to further exclusion of donor tissues. About 9% of all cultured corneas in Germany have to be discharged due to contamination [15].

Far-UVC, with a spectral range of 200–230 nm [16], exhibits strong antimicrobial properties similar to the antimicrobial properties at 254 nm UVC radiation, based on DNA and RNA absorption [17]. Far-UVC is also absorbed by proteins, which largely shield the nucleus in irradiated mammalian cells [18,19]. Studies have shown that Far-UVC doses up to 600 mJ/cm^2^ are harmless to the outer corneal layers, while a dose of 15 mJ/cm^2^ is sufficient for a 3 log reduction in methicillin-resistant *Staphylococcus aureus* (*S. aureus*) (MRSA) [20,21,22,23]. The American Conference of Governmental Industrial Hygienists (ACGIH) sets the safe exposure limit for eyes to 222 nm Far-UVC irradiation at 167.6 mJ/cm^2^ [24].

The hCorneas are retrieved from deceased donors in a sterile environment, following strict eye bank protocols to preserve sterility and tissue viability while minimizing damage during dissection [25]. After extraction, corneas are disinfected to eliminate pathogens, typically by immersion in 3% povidone-iodine, followed by saline rinsing [26]. In addition, atmospheric-pressure cold plasma (APCP) has been explored as an alternative disinfection method, effectively inactivating pathogens without compromising tissue integrity [27]. For xenotransplantation, the extraction of porcine corneas typically begins with anesthesia to ensure the animal remains still and pain-free [28]. The eyes are then enucleated in a sterile environment to prevent contamination, followed by precise dissection of the cornea to minimize tissue damage [29]. Once extracted, the corneas are preserved in an appropriate medium to maintain viability for research or transplantation purposes [30].

The objective of this study is to determine the appropriate disinfecting dose of 222 nm Far-UVC radiation that can be tolerated by CECs while being sufficient to induce decontamination. For this purpose, the human corneal endothelial cells (hCECs) of cultured human corneas (hCorneas) are irradiated with up to 60 mJ/cm^2^, cultured again, and the cell density is recorded at regular intervals. A set of cultured corneas is used to compare the behavior during recultivation after irradiation. Furthermore, pairs of eyes from the same donor are subjected to an investigation to ascertain the existence of any potential correlations. Experiments on porcine corneas (pCorneas) at irradiation doses of up to 150 mJ/cm^2^ on porcine corneal endothelial cells (pCECs) provide supplementary evidence. The results are subjected to statistical evaluation.

## 2. Materials and Methods

The pCorneas used in this study were obtained from freshly slaughtered pigs. The corneas were extracted from the bulbi in a sterile laboratory environment. Prior to extraction, the bulbi were disinfected in a 2% iodine solution for three to five minutes and subsequently washed in sterile balanced salt solution (BSS). Extraction was performed by cutting along the scleral rim. The extracted corneal disc was temporarily stored in sterile phosphate-buffered saline (PBS).

The hCorneas were provided by the tissue bank IKT (Institute for Clinical Transfusion Medicine and Immunogenetics) Ulm, Germany. The cell densities at the beginning and end of the cultivation process were recorded. A set comprising corneas routinely cultivated in the tissue bank was employed to assess the normal performance of the corneas during cultivation and to ascertain the independence of two corneas from the same donor.

The corneas of humans and pigs are structurally similar in diameter (10–12 mm vs. 12–14 mm) and thickness (550–700 µm vs. 666–1013 µm). There is a similarity in the density of CECs (2500–4000 cells/mm^2^ in the hCorneas and 3250–4411 cells/mm^2^ in the pCorneas) and in the layers (epithelium, Bowman’s membrane, stroma, Descement’s membrane and endothelium) [13,29,31]. The mechanical properties are comparable in tensile strength and stress–strain relationships under uniaxial testing, but there are significant differences in stress relaxation [30]. The pCECs recover or regenerate more than hCECs, which mostly do not. This characteristic contributes to the resilience of pCECs for transplantation, making it more likely that, with appropriate therapy, porcine tissues can be applied in medical treatments [29]. The probability of an immune response arising from Far-UVC exposure to the endothelium is negligible because the primary objective is to avert any potential damage to the CECs.

**Figure 1:**
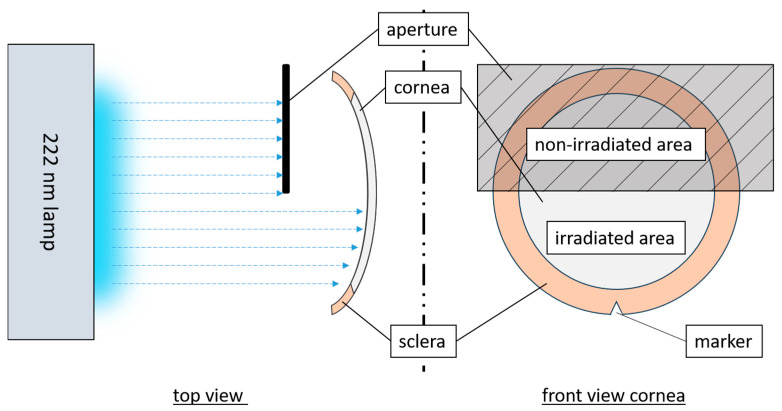
Schematic representation of the irradiation setup for hCornea with the partially blocked radiation pathway (top view) and the irradiation areas of a cornea (front view).

The irradiation setup used a krypton chloride excimer lamp (Care222^®^, Ushio, Tokyo, Japan), and an X1 optometer (Gigahertz-Optik, Türkenfeld, Germany) was used to set an irradiance of 1 mW/cm^2^. The cornea was fixed in a Böhnke holder within an empty modified cell culture flask with a window for the irradiated area (Figure 1). The concave side of the cornea with the CECs was irradiated, in comparison to other tests (given that the cornea is not transparent at 222 nm [32,33,34]). In hCornea experiments, one half was irradiated, and the other served as control. To distinguish between sides, an incision was made at the scleral edge on each sample. The non-irradiation area was adequately covered. The pCECs were fully irradiated at doses of 60 and 150 mJ/cm^2^ while hCECs received half-sided irradiation of 15 and 60 mJ/cm^2^.

To examine the pCornea microscopically, an irradiated sample was trephined after a latency period of up to two hours. This corneal section was then stained. Viable CECs were evaluated and compared through staining in accordance with the instructions provided in the Viability/Cell toxicity Assay Kit for Animal Live & Dead cells (Biotium, Fremont, CA, USA) or with DAPI (4′,6-diamidino-2-phenylindole, dilactate) (Invitrogen, Carlsbad, CA, USA). An Eclipse TE2000-U (Nikon, Gyoda, Japan) fluorescence microscope was used to obtain multiple images of each cornea.

The integrity and density of the hCECs were determined via cell imaging prior to and immediately following irradiation, as well as at regular intervals up to the fifth day. Therefore, hCorneas were placed in a chamber of a 6-well plate, which was filled with 10 mL sterile PBS. At least five images of the hCECs per corneal half (irradiated/non-irradiated) were taken at each time of measurement with a phase contrast microscope (Primovert, Zeiss, Oberkochen, Germany) with 40× magnification.

The evaluation of pCECs and hCEC densities was carried out with ImageJ 1.53o [35] by creating a grid with a size of 0.1 × 0.1 mm^2^ over the images to be analyzed. By using the CellCounter plugin [36], the cells of 4 fields were counted and averaged. This average was multiplied by one hundred to obtain cells/mm^2^.

A disinfection test was conducted using *Staphylococcus carnosus* (DSM 20501) as a surrogate for *S. aureus*, given its comparable sensitivity to 222 nm UVC radiation [37]. A colony of *S. carnosus* was inoculated into 3 mL M92 medium and incubated at 37 °C for 16 h. Then, 200 µL of the preculture was transferred to 30 mL of fresh M92 medium and incubated at 37 °C until an optical density at 600 nm (OD_600_) of 0.33 was reached. The culture was centrifuged and the supernatant replaced with PBS. This was repeated twice. The suspension was diluted to 10⁷ colony-forming units (CFU) per milliliter. Disinfection tests were conducted on the concave side of pCorneas using two different thicknesses of bacterial suspension (1 mm and 3 mm) and three different Far-UVC doses (15, 60, and 150 mJ/cm^2^). Following this, 33 µL samples were plated on M92 agar and incubated at 37 °C for 24 h to determine bacterial survival.

To analyze changes in cell density of hCECs over a five-day period, the cell density ratios (CDRs) were calculated by dividing the value of the last cell count by the value of the first cell count (Equation (1)) for each sample. It was assumed that a five-day observation period would be sufficient for the human corneas to show an effect on the sensitive endothelium.

(1) $$cell\;density\;ratio\;(CDR)=\frac{{cells/{mm}^{2}}_{end\;of\;cultivation}}{{cells/{mm}^{2}}_{start\;of\;cultivation}},$$

The relative change of cell counts (RCCC) was calculated by dividing CDR of irradiated side by CDR of non-irradiated side (Equation (2)).

(2) $$RCCC=1-\frac{{CDR}_{irr}}{{CDR}_{non-irr}}=1-\frac{{day5}_{irr}/{day1}_{irr}}{{day5}_{non-irr}/{day1}_{non-irr}},$$

Initial data analysis involved assessing normality using the Shapiro–Wilk test. To evaluate the homogeneity of variances, the Levene test was conducted. When variance homogeneity was confirmed, analysis of variance (ANOVA) with the F-test for multiple samples was performed. In the case of significant ANOVA results, the Bonferroni correction was applied to adjust the significance levels. If the data were not normally distributed, the Kruskal–Wallis test (H-test) was employed. For significant results from the Kruskal–Wallis test, Dunn’s post hoc test was subsequently performed. A *p*-value of ≤ 0.05 was considered statistically significant. A Student’s *t*-test for unpaired samples was conducted to ascertain the statistical significance of the observed differences. Analyses were performed using the statistical software R 4.2.1 [38].

Based on an assumed effect δ and standard deviation (SD), the sample size was determined in order to have 80% power (1-ß = 80% with a corresponding $z_{1-\beta }$-quantile of 0.84) for a significance level of 5% (corresponding z1−α2-quantile of 1.96) using Equation (3) [39].

(3) n=z1−α2+z1−β2∗ SD2δ2,

SD was calculated according to Equation (4). In order to calculate δ according to Equation (5), the initial test results were employed to derive the mean values.

(4) $$SD=\sqrt{\frac{\sum {\left(x-\bar{x}\right)}^{2}}{n-1}}$$

(5) $$\delta =\left({µ}_{1}-{µ}_{0}\right)$$

A reference group (RG) was established for the purpose of determining normal behavior and for test size analysis. The reference data were based on 168 transplantable corneas from the tissue bank IKT Ulm.

The study size was calculated using the SD of the RG, as this group had sufficient values and represents normal behavior. The δ between the irradiated and non-irradiated corneal sides of initially irradiated hCornea samples was calculated and used.

(6) $${SD}_{RG}=\sqrt{\frac{\sum {\left(x-\bar{x}\right)}^{2}}{n-1}}=0.081$$

(7) $$\delta_{hC-sample}=\left(0.964-0.916\right)=0.048$$

(8) $$n={\left(1.96+0.84\right)}^{2}*\;\frac{0.081^{2}}{0.048^{2}}=22.3;\;n=23$$

The RG has an average hCEC density of 2510 cells/mm^2^, with an SD of 244 cells/mm^2^. By using a δ of 5% of the mean, a comparable sample size can be obtained:

(9) $$n={\left(1.96+0.84\right)}^{2}*\;\frac{244^{2}}{125.5^{2}}=29.6;\;n=30$$

## 3. Results

This study evaluated the applicability of an effective disinfection dose without compromising endothelial integrity.

The irradiation of the hCECs was conducted within a room exclusively designated for laboratory examinations. The removal times of the cornea from the culture medium or buffer were maintained as short as possible to ensure the preservation of tissue integrity, with only minor extensions being made to extend the removal times for the irradiation process. The porcine corneas were processed in a laboratory and subsequently handled with minimal exposure outside of the buffer. All experiments were conducted at room temperature and under aseptic conditions.

Irradiation was applied to the concave corneal side, the critical surface for donation, due to the endothelium’s sensitivity and lack of regenerative capacity. The cornea’s concave–convex shape led to slight inhomogeneity in surface irradiation; however, with central alignment, intensity variation remained within 0.08 mW/cm^2^. At a lamp-sample distance of ~9 cm, no heating occurred.

### 3.1. pCornea Results

**Figure 2:**
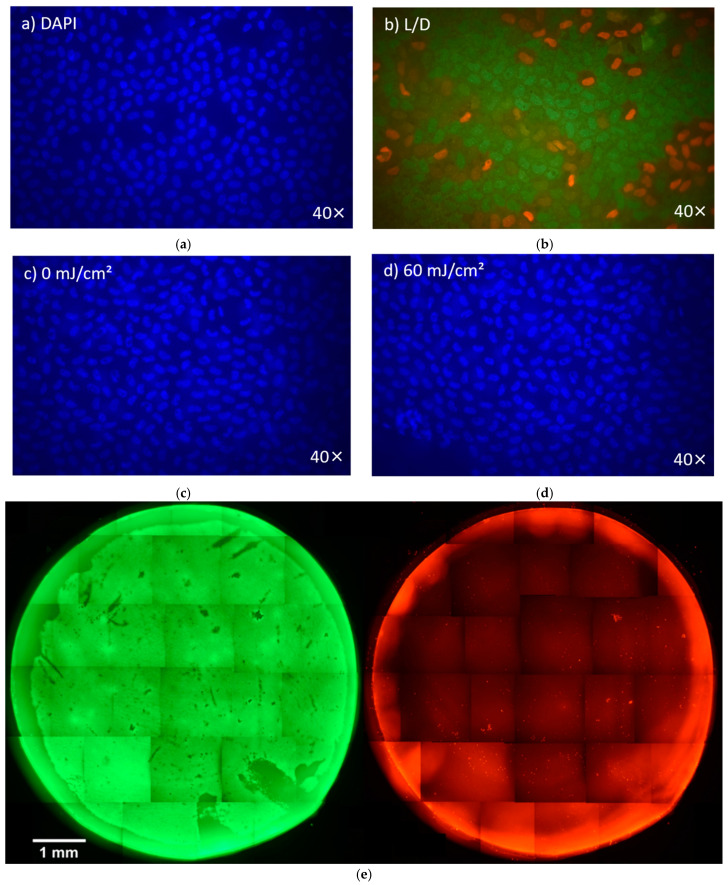
Comparison between a DAPI stain (**a**) and a live–dead stain (**b**); comparison between a non-irradiated (**c**) and 60 mJ/cm^2^ irradiated endothelial layer after 2 days of cultivation. Images taken with a fluorescence microscope at 40× magnification (**d**). A disc of cornea was cut from a pCornea and stained with a live–dead dye. The two stains, live (left, green) and dead (right, red), are recorded separately from the same cornea. Images taken with a fluorescence microscope at 10× magnification and stitched together (**e**).

Staining results demonstrated accurate and clear cell nuclei in both DAPI and live–dead staining using fluorescence microscopy. There was no noticeable density thinning or consistent decrease in density. However, in DAPI detection, there were areas where no CECs were present, as shown in Figure 2a. The staining for live and dead CECs revealed damaged or dead CECs in these areas (Figure 2b). A comparison of the two staining methods revealed no significant deviation in either variance (*p* = 0.135) or mean (*p* = 0.115). These findings support the applicability of DAPI staining for determining cell density. Structured damage caused by processing during trephination and dying, such as furrows, tweezer marks, and other pressure points, was not included in the count (Figure 2e). Additionally, irregular accumulations of damaged CECs were occasionally observed, as illustrated in Figure 2b. The cell shapes and spacing of the stained CECs showed an equal and regular distribution (Figure 2a–d). The staining of pCECs after 222 nm irradiation up to 150 mJ/cm^2^ did not lead to a loss of structural integrity.

The irradiation tests revealed that the mean CEC densities of the irradiation groups listed in Table 1 are distributed from 3873 to 3414 cells/mm^2^. The SDs are around 9%, with only a few outliers. None of the outliers are below 3000 cells/mm^2^, but some are above 4500 cells/mm^2^. These outliers were included due to their high CEC densities, which are still within the characteristic range [29].

**Figure 3:**
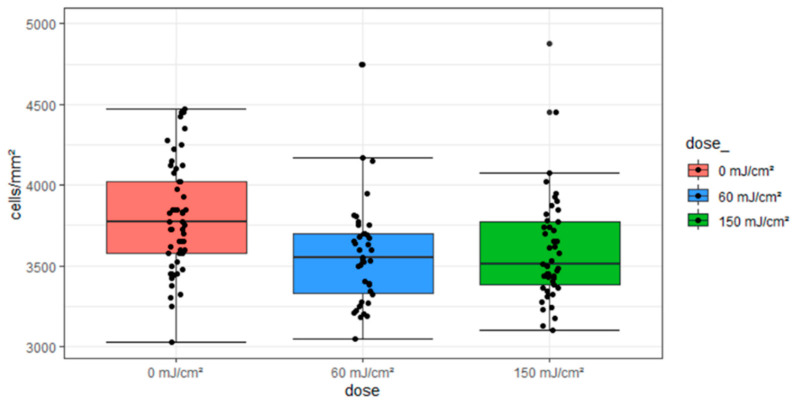
Dose-dependent grouping of pCEC densities from 222 nm irradiation.

The box plots representing the groups are presented in Figure 3.

In order to compare the three irradiation doses regarding the CEC densities, the Kruskal–Wallis test was applied since normality could not be assumed (demonstrated with Shapiro–Wilk test) and outliers are present in the sample. This Kruskal–Wallis test is more robust compared to a conventional ANOVA.

The Kruskal–Wallis test (χ^2^(2) = 14.87, *p* < 0.001) and post hoc tests demonstrated significant differences between the 0 mJ/cm^2^ and 60 mJ/cm^2^ groups and between the 0 mJ/cm^2^ and 150 mJ/cm^2^ groups.

**Figure 4:**
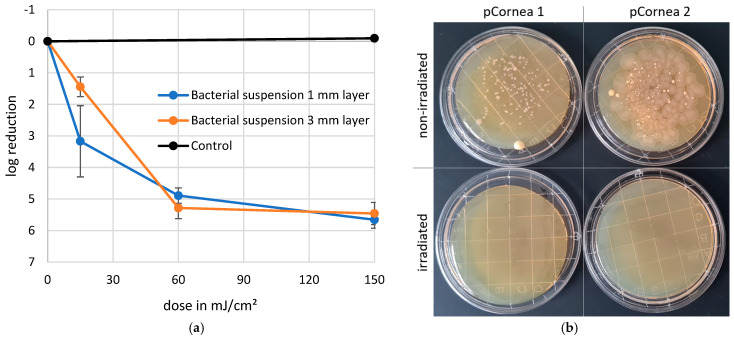
222 nm disinfection of a one- and three-millimeter-thick layer of an *S. carnosus* bacterial suspension in a log-reduction graph (**a**) and a picture of contamination samples before and after irradiation plated on a tryptone soy agar plate to demonstrate the efficacy of the process (**b**).

The results of the microbiological (Figure 4) tests demonstrated that irradiation doses of 15 mJ/cm^2^ achieved up to a 3-log reduction in microbial load within 1 mm thick liquid films. Furthermore, reductions of up to and over 5 log levels were achieved in 1 and 3 mm liquid layers with 60 mJ/cm^2^.

### 3.2. hCornea Results

**Figure 5:**
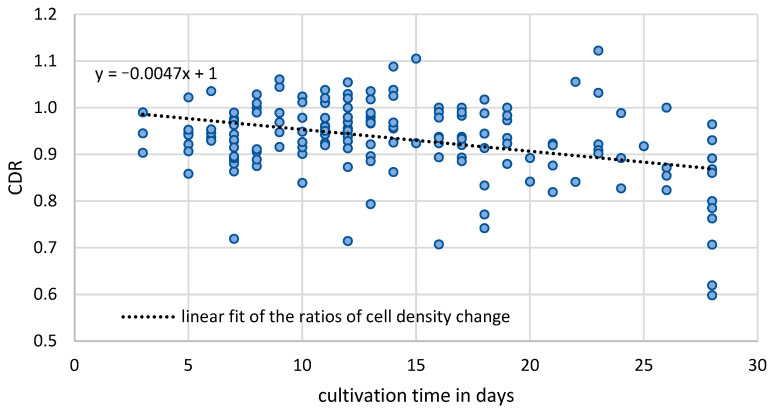
CDR values of the referenced group of the set of cultured corneas from the IKT Ulm, Germany.

The mean hCEC density of the RG on the first day of culture was 2509 cells/mm^2^ (SD = 243 cells/mm^2^). On the last day (3 to 28 days later), the mean was 2338 cells/mm^2^ (SD = 283 cells/mm^2^). The mean CDR between the first and last day was 0.93 (SD = 0.081). Figure 5 presents all CDRs of the referenced data as a function of cultivation time, with a slightly decreasing straight line fit (CDR = −0.0047x + 1) (approx. 0.5% loss per day) for cultivation durations of 3 to 28 days (*n* = 168). The Shapiro–Wilk test was *p* < 0.001, indicating non-normal distribution of the CDRs, but the large sample size mitigates this issue for the regression model.

Verification of the correlation between two corneas from the same donor: A total of 26 corneal pairs from the RG were analyzed, exhibiting a linear regression for the right/left ratio with a low coefficient of determination (R^2^ = 0.0232) and no consistent or trend-like distribution of residuals. The regression lines for both corneal sides were parallel with a slight CDR offset of 0.025, indicating no significant difference in hCEC density behavior. A *t*-test confirmed this (*p* = 0.491).

Results of the irradiation tests: The normal incubation period of the corneas was five days, but one cornea was only incubated for three days as the quality of the hCECs deteriorated rapidly and could not be counted later. The hCEC densities from day five and the outlier from day three were used for statistical analysis. There was no visible change in the culture medium in any of the tests.

The CDR differences between the irradiation groups in Table 2, comprising irradiated vs. non-irradiated samples at each dose, resulted in a deviation of approximately 4.5% at 15 mJ/cm^2^ and 5% at 60 mJ/cm^2^. A two-sample *t*-test was conducted on the irradiation groups 15 (*p* = 0.667) and 60 mJ/cm^2^ (*p* = 0.582), in which the CDR values of the non-irradiated corneal halves were compared against the corneal halves within the group. Nevertheless, no evidence of significance could be provided.

A comparison of the CDRs of the non-irradiated samples, designated “non-irradiated 15” and “non-irradiated 60”, revealed no evidence for a statistically significant difference (t(9) = −0.101, *p* = 0.922). A corresponding calculation of the Cohen’s d with the CDRs of the irradiation results indicated a tendency towards a small effect, with d = 0.308.

**Figure 6:**
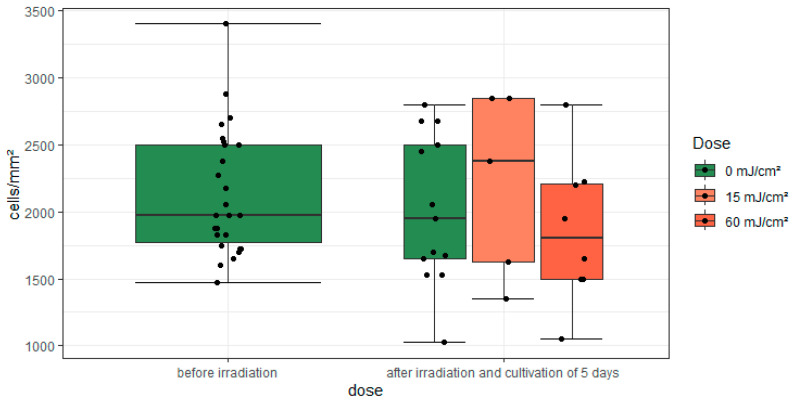
A grouped boxplot of the cell density depending on the irradiation dose.

An ANOVA was conducted with the CDRs for each dose (0, 15, and 60 mJ/cm^2^), revealing that the difference between the sample means of all groups in the comparison of irradiation application was not statistically significant (F(2,23) = 0.273, *p* = 0.764, η^2^ = 0.02) (Figure 6, boxes on the right side). To assess the equality of variances, the Levene test was performed, which confirmed the assumption of equal variances (*p* = 0.919).

A further summary was conducted for the irradiated samples labelled ‘irradiated 15’ (*n* = 5) and ‘irradiated 60’ (*n* = 8), for which no statistically significant result could be demonstrated (*p* = 0.954). The data from the irradiated and non-irradiated samples were, therefore, summarized as 13 values each. A two-sample *t*-test of all irradiated and non-irradiated sample sides yielded a *p*-value of 0.462. These results suggest that the different doses did not have a measurable effect on the CDRs, indicating a lack of efficacy in this context.

**Figure 7:**
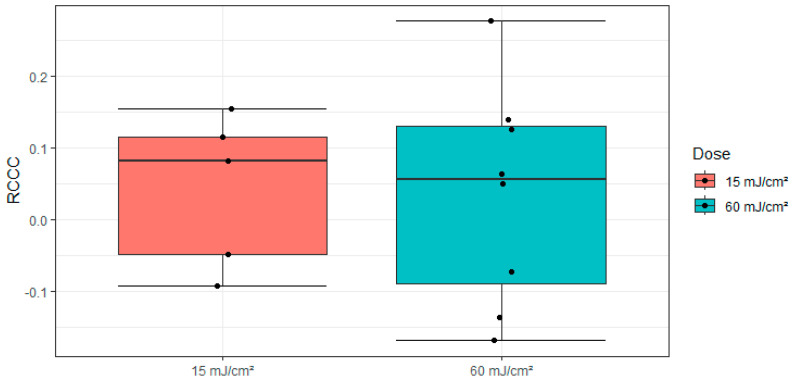
Boxplot of the RCCC values (dots) with the division into the irradiation groups 15 mJ/cm^2^ (orange) and 60 mJ/cm^2^ (blue).

Calculation of the RCCC, shown in Figure 7, and the cell count decrease between the first and the last day: The mean RCCC values were 0.042 for the 15 mJ/cm^2^ group and 0.035 for the 60 mJ/cm^2^ group, with an overall mean of 0.037. A *t*-test revealed no significant difference between the groups (t(11) = 0.091, *p* = 0.929, ƞ^2^ = 0.056, d = 0.051). Both groups were normally distributed (15 mJ/cm^2^: *p* = 0.531, 60 mJ/cm^2^: *p* = 0.844).

## 4. Discussion

### 4.1. pCorneas

The CEC staining procedures did not yield any discernible alterations to the endothelium as a consequence of the radiation treatment. Consequently, any potential impairment in structural integrity can be ruled out.

The SD for the three test samples (0, 60 and 150 mJ/cm^2^) was approximately 9%, indicating a similar degree of variability amongst the data points. The results of the Shapiro–Wilk test indicated that the 60 mJ/cm^2^ and 150 mJ/cm^2^ samples deviated from a normal distribution. Additionally, the groups exhibited outliers, making ANOVA insufficiently robust despite sufficient sample sizes of 57, 39, and 45. Therefore, the Kruskal–Wallis test was applied instead. The 60 and 150 mJ/cm^2^ groups exhibited left skewness, likely due to outliers at high CEC densities, and resulted in a significant difference among the three samples. Nonetheless, the samples generally showed similar ranges, with means differing by less than 10%. Additionally, the evaluation did not indicate a significant difference in the applied exposures, suggesting a lack of effect rather than an actinic effect. However, further investigation is required into the impact of the effects on the subjects, as additional factors such as the animals’ origins, handling of the subjects, and the processing of the subjects may also be contributing to the results.

Given the natural variability in CEC density among corneas, using a ratio metric such as the CDR or RCCC would have been more appropriate for analyzing and assessing the resulting cell densities post-irradiation with Far-UVC. This approach would have allowed for tracking changes in CEC density over time for each sample, facilitating better comparisons with other samples. Comparisons between hCECs and pCECs showed similar dispersion patterns and standard deviations.

The irradiation of the bacterial suspension on the endothelium was a relatively simple experimental setup, but it was quantitatively suboptimal. The high bacterial concentration on the endothelial surface represented an extreme case of microbial contamination, far from typical initial contamination levels. Nonetheless, the disinfection test clearly demonstrated the potential efficacy and application scope of this method.

### 4.2. hCorneas

The results of the paired comparisons suggest that a correlation between the CEC densities in the right and left corneas of a donor can be excluded and that all usable samples, including both corneas from the same donor, can be considered independent.

The CDR values of the entire reference group exhibit a regression pattern similar to that of the non-irradiated CECs in the test group during extended cultivation. The comparable slopes of the linear regressions in both groups suggest that their temporal behavior is nearly identical. The model indicates that while irradiation affects CEC density, this effect should be considered in the context of the normal cellular loss associated with cultivation.

The absence of a notable CDR test difference with the ANOVA test (*p* = 0.767) and the small effect size (η^2^ = 0.02) lead to the conclusion that the irradiation is applicable. However, a significant alteration in CEC density could not be substantiated.

The relative difference in RCCC values between the two radiation doses of 0.007 is small compared to the mean value (0.037), as confirmed by a *t*-test (*p* = 0.923). The mean RCCC value of 0.037 corresponds to a 3.7% reduction in cell density in irradiated CECs compared to non-irradiated cells. This small effect size supports the hypothesis of a minimal radiation impact. It is important to note that the sample sizes for the RCCC groups (*n* = 5 and *n* = 8) are insufficient for precise study dimensioning but may be adequate for indicating trends.

### 4.3. Overall

Far-UVC at 222 nm has been shown to inactivate a wide range of bacteria, including those that are resistant to conventional antibiotics. This efficacy has also been demonstrated through the microbial test with S. carnosus as a surrogate for MRSA (see Figure 4) [37]. In contrast to conventional UVC, Far-UVC does not cause damage to cell nuclei or DNA due to its high protein absorption [40], making it a suitable method for surface disinfection and contamination control in clinical settings [41] on biological tissues and inorganic surfaces. However, certain limitations must be considered. Due to the shallow penetration depth of the radiation, its effectiveness is primarily restricted to surface disinfection or areas in close proximity to the exposure source. As a result, Far-UVC lacks the ability to penetrate deeper into tissues or complex structures, limiting its applicability in certain scenarios. These characteristics should be carefully evaluated when implementing this technology in clinical or industrial environments.

Due to the limited availability of hCorneas used in this study, the findings were supplemented by comparative investigations on porcine corneas. Although the initial results did not indicate any fundamental actinic changes in the CEC, it would be advisable to extend the studies on human corneas to the required sample size. Furthermore, additional studies should be conducted to obtain reliable conclusions. In particular, these should include investigations on the effects of increased radiation doses, assessments of cellular damage, and analyses of cellular changes following long-term incubation for up to 30 days.

On a structural level, there is similarity between human and porcine corneas. Combined with the apparent translatability, a similar response to environmental insults could be expected [29,30]. However, immunological disparities may influence responses to radiation, necessitating further investigation to assess their stability and suitability for exposure to radiation [29]. However, it is worth noting that the application of Far-UVC irradiation was limited to the CECs, because deeper tissue layers were not penetrated as the penetration depth was simply too low due to the high absorption [40].

## 5. Conclusions

Overall, no definitive conclusions could be drawn regarding the different test groups for porcine corneas due to excessive variance. The comparison based on cell densities without adjusting for individual variances proved problematic.

In contrast, when using CDR values and the resulting normalized values for human corneas, no significant effects from exposure to 222 nm radiation at doses up to 60 mJ/cm^2^ were observed. This suggests that using 222 nm radiation for disinfection does not compromise the integrity of the endothelium, although it should be noted that the sample size was not sufficient for definitive conclusions. Further investigation into the threshold at which 222 nm radiation causes damage would be valuable. However, longer exposure times outside of a fluid buffer are unsuitable for the cornea. Irradiation within a non-absorbing buffer, such as PBS or BSS, could be considered.

## Figures and Tables

**Table 1:** The pCEC densities after irradiation at 222 nm, divided into the applied irradiation dose.

Irradiation Dose [mJ/cm^2^]	0	60	150
Mean CECs [cells/mm^2^]	3798	3572	3601
SD [cells/mm^2^]	337	320	335
Number of corneas	57	39	45

**Table 2:** The mean values ± SD of the results of the experiments with and without irradiation of hCECs, along with their CDRs (cell density ratios) of the cell densities from the day of irradiation and five days later, as well as the resulting RCCCs (relative change of cell counts).

		15 mJ/cm^2^	60 mJ/cm^2^	Σ
	n	5	8	13
Mean CEC counts for irradiated corneas ± standard deviation and cell density ratio (CDR)	day1	2405 ± 593	2028 ± 351	2173 ± 494
	day5	2205 ± 626	188 ± 521	2008 ± 585
	CDR	0.913	0.918	0.916
Mean CEC counts for non-irradiated corneas ± standard deviation and cell density ratio (CDR)	day1	2270 ± 450	1991 ± 350	2098 ± 414
	day5	2180 ± 539	1931 ± 511	2027 ± 535
	CDR	0.958	0.968	0.964
	RCCC	0.042	0.035	0.037

## Data Availability

The original contributions presented in this study are included in the article. Further inquiries can be directed to the corresponding author.
